# Hypertension Control and Cardiometabolic Risk: A Regional Perspective

**DOI:** 10.1155/2012/925046

**Published:** 2012-01-02

**Authors:** Martin Thoenes, Peter Bramlage, Sam Zhong, Shuhua Shang, Massimo Volpe, David Spirk

**Affiliations:** ^1^Institute for Clinical Pharmacology, Medical Faculty Carl Gustav Carus, Technical University Dresden, 01307 Dresden, Germany; ^2^Léman Research Institute GmbH, 6300 Zug, Switzerland; ^3^Institute for Cardiovascular Pharmacology and Epidemiology, 15831 Mahlow, Germany; ^4^Medical Affairs Department, Sanofi, Shanghai 200000, China; ^5^Cardiology Division and Hypertension Unit, S. Andrea Hospital, La Sapienza University of Rome, 00189 Rome, Italy; ^6^I. R. C. C. S. Neuromed, 86077 Pozzili, Italy; ^7^Medical Affairs Department, Sanofi-Aventis (Suisse) SA, 1217 Meyrin, Switzerland

## Abstract

*Background*. We investigated the association between blood pressure control and common cardiometabolic risk factors from a global and regional perspective. *Methods*. In the present analysis of a large cross-sectional i-SEARCH study, 17.092 outpatients receiving antihypertensive treatment were included in 26 countries. According to clinical guidelines for the management of arterial hypertension, patients were classified based on the level of seated systolic/diastolic blood pressure (SBP/DBP). Uncontrolled hypertension was defined as SBP/DBP ≥140/90 mmHg for non-diabetics, and ≥130/80 mmHg for diabetics. *Results*. Overall, mean age was 63.1 years, 52.8% were male, and mean BMI was 28.9 kg/m^2^. Mean SBP/DBP was 148.9/87.0 mmHg, and 76.3% of patients had uncontrolled hypertension. Diabetes was present in 29.1% with mean HbA1c of 6.8%. Mean LDL-cholesterol was 3.2 mmol/L, HDL-cholesterol 1.3 mmol/L, and triglycerides 1.8 mmol/L; 49.0% had hyperlipidemia. Patients with uncontrolled hypertension had a higher BMI (29.4 versus 28.6 kg/m^2^), LDL-cholesterol (3.4 versus 3.0 mmol/L), triglycerides (1.9 versus 1.7 mmol/L), and HbA1c (6.8 versus 6.7%) than those with controlled blood pressure (*P* < 0.0001 for all parameters). *Conclusions*. Among outpatients treated for arterial hypertension, three quarters had uncontrolled blood pressure. Elevated SBP/DBP and uncontrolled hypertension were associated with increasing BMI, LDL-cholesterol, triglycerides, and HbA1c, both globally and regionally.

## 1. Introduction

Arterial hypertension represents a major cause of cardiovascular morbidity and mortality, and affects approximately 1 billion individuals worldwide [1, 2]. Despite the availability of efficient nonpharmacological and pharmacological therapies, blood pressure control rates are largely unsatisfactory, mostly due to underdiagnosis and undertreatment [3]. Furthermore, arterial hypertension is frequently clustered with other metabolic disorders, such as an elevated body mass index (BMI), waist circumference (WC), fasting glucose, triglycerides (TG), and HDL-cholesterol—all of which are associated with adverse cardiovascular outcomes [4–7]. Therefore, international guidelines mandate not only an assessment of the global cardiovascular risk, but also a risk-based approach to antihypertensive therapy [8]. Apart from the impact of the association of an elevated blood pressure with metabolic disorders on patient's cardiovascular risk, there are also implications from a therapeutic perspective. Recent data have shown independent antihypertensive effects of statins in patients with hypertension and hypercholesterolemia, and an association of blood pressure lowering with a decrease in the antioxidative activity of HDL-cholesterol [9, 10]. These data illustrate not only a potential cross-talk between different biochemical pathways, involved in the pathogenesis of atherosclerotic disease, but also the ability of pharmacological treatments to act on several risk factors at the same time. Especially in light of the low blood pressure control rates worldwide, it appears to be important to have a deeper understanding of the association of blood pressure with relevant metabolic risk factors and cardiovascular risk markers. The present analysis aims to investigate the association of blood pressure control with several metabolic risk factors/cardiovascular risk indicators, and to gain insights into regional/ethnical differences of these associations from a large international survey, conducted in more than 20.000 patients with arterial hypertension.

## 2. Methods

A large cross-sectional International Survey Evaluating microAlbuminuria Routinely by Cardiologists in patients with Hypertension (i-SEARCH) study was conducted in 2005-2006 in cardiology outpatient clinics in 26 countries world-wide as described previously [11]. 21.794 patients, aged ≥18 years with currently treated or newly diagnosed arterial hypertension, were enrolled into the study. In all patients, urinary dipstick screening was performed and the prevalence of microalbuminuria (MAU) was determined. Furthermore, information on patient demographics, anthropometric measures, cardiovascular risk factors, metabolic parameters, comorbid conditions, and cardiovascular drug therapy was collected. The present analysis was performed in 17.092 patients receiving antihypertensive treatment. According to contemporary clinical guidelines for the management of arterial hypertension [8], patients were classified based on the level of seated systolic/diastolic blood pressure (SBP/DBP) measured at rest on day of study visit. For each level of SBP (<120, 120-139, 140-159, 160-179, ≥180 mmHg) and DBP (<90, 90-99, 100-109, ≥110 mmHg), the association with the following indicators of cardiometabolic risk was determined: BMI (kg/m^2^), WC (cm), diabetes mellitus (%), HbA1c (%), LDL/HDL-cholesterol (mg/dL), triglycerides (mg/dL), and C-reactive protein (CRP, mg/dL). Furthermore, cardiometabolic risk was determined according to blood pressure control. Uncontrolled hypertension was defined as SBP/DBP ≥140/90 mmHg for non-diabetic and ≥130/80 mmHg for diabetic patients.

All analyses were performed both globally and separately for the following 5 geographical regions: Northern Europe (Belgium, Germany, Sweden, Switzerland), Southern Europe (Greece, Italy, Spain, Turkey), North America (Canada), Middle East (Kuwait, Lebanon, Qatar, Saudi Arabia, United Arab Emirates) and Asia (Hong Kong, Indonesia, Korea, Singapore, Taiwan, Thailand, Vietnam). The analysis population comprised patients with no missing data for SBP/DBP and the respective metabolic parameter. A linear model was used to estimate the least square means of BMI, WC, HbA1c, LDL-cholesterol, HDL-cholesterol, triglycerides, and CRP for each level of SBP/DBP and by region. The model was adjusted for age and gender (BMI, WC, and CRP); for age, gender, and antidiabetic treatment (HbA1c); and for age, gender, and the presence of diabetes (LDL- and HDL-cholesterol, triglycerides). A logistic regression analysis was conducted to estimate the prevalence of diabetes for each level of SBP/DBP and region, adjusted for age and gender (predictive marginal probabilities). Continuous variables are depicted as adjusted means (least square means) ± standard deviations and categorical variables as percentages (95% confidence intervals).

## 3. Results

### 3.1. Cardiometabolic Risk Profile

Overall, mean patient age was 63.1 years out of which 52.8% were male. Mean SBP/DBP was 148.9/87.0 mmHg, and 76.3% of patients had uncontrolled blood pressure. Diabetes was present in 29.1% of patients with mean HbA1c of 6.8%. Mean LDL-cholesterol was 3.2 mmol/L, mean HDL-cholesterol 1.3 mmol/L, and mean triglycerides 1.8 mmol/L, and 49.0% of patients had hypercholesterolemia. MAU was present in 58.8% of patients, and mean CRP was 0.92 mg/dL. 38.8% of patients were present or past smokers, and 28.6% had a family history of a myocardial infarction. For concomitant cardiovascular disease and regional distribution of individual parameters, see Table 1.

### 3.2. Blood Pressure and BMI/WC

Globally, the mean BMI was higher in patients with SBP ≥180 versus <120 mmHg (29.5 versus 28.2 kg/m^2^), in patients with DBP ≥110 versus <90 mmHg (30.3 versus 28.5 kg/m^2^), and in patients with uncontrolled versus controlled blood pressure (29.4 versus 28.6 kg/m^2^) (*P* < 0.0001 for all parameters). Mean WC was higher in patients with SBP ≥180 versus <120 mmHg (101.2 versus 97.5 cm), in patients with DBP ≥110 versus <90 mmHg (103.2 versus 98.8 cm), and in patients with uncontrolled versus controlled blood pressure (100.7 versus 98.8 cm) (*P* < 0.0001 for all parameters). By comparing the association of BMI and WC across the regions, an increase in BMI with increasing SBP/DBP could be observed for Northern, Southern Europe and the Middle East region, whereas in North America and Asia, BMI decreased with increasing SBP, and increased with DBP (*P* < 0.05 for all comparisons). Only in Northern and Southern Europe, uncontrolled versus controlled blood pressure was associated with an increase in BMI (*P* < 0.0001). With increasing SBP/DBP an increase in WC could be observed for Northern Europe, Southern Europe, North America, and Middle East (in the latter only for DBP, *P* < 0.0001), whereas an inverse relationship between SBP/DBP and WC was observed for Asia (*P* < 0.0001). For details see Tables 2 and 3.

### 3.3. Blood Pressure and Diabetes/HbA1c

The prevalence of diabetes was 28.4% in patients with an SBP <120 mmHg and 32.6% in patients with an SBP ≥180 mmHg (*P* < 0.0001). Diabetes was present in 30.9% of patients with a DBP <90 mmHg and 28.1% of patients with a DBP ≥110 mmHg (*P* < 0.0001). There was no difference in the prevalence of diabetes in patients with uncontrolled versus controlled hypertension in the overall population (27.7% versus 30.4%; *P* = 0.18). Mean HbA1c increased from 6.7% in patients with an SBP of <120 mmHg to 7.0% in patients with an SBP of ≥180 mmHg (*P* < 0.0001), from 6.8% in patients with a DBP <90 mmHg to 6.9% in patients with a DBP ≥110 mmHg (*P* < 0.0027), and from 6.7% in patients with controlled to 6.8% in patients with uncontrolled blood pressure (*P* < 0.0001). A significant increase in HbA1c with SBP and DBP was observed in Northern and Southern Europe, but not in Northern America, Middle East, and Asia. For details, see Tables 4 and 5.

### 3.4. Blood Pressure and Lipids

The mean LDL-cholesterol was higher in patients with SBP ≥180 versus <120 mmHg (3.4 versus 2.9 mmol/L), in patients with DBP ≥110 versus <90 mmHg (3.5 versus 3.0 mmol/L), and in patients with uncontrolled versus controlled blood pressure (3.4 versus 3.0 mmoL) (*P* < 0.0001 for all parameters). Mean HDL-cholesterol was 1.3 mmol/L, and there was no association between HDL in patients with uncontrolled versus controlled hypertension (*P* = 0.13). Triglycerides increased from 1.5 mmol/L in patients with an SBP <120 mmHg to 1.9 mmol/L in patients with an SBP ≥180 mmHg, and from 1.7 mmol/L in patients with a DBP <90 mmHg to 1.9 mmol/L in patients with a DBP ≥110 mmHg (*P* < 0.0001 for both parameters). Triglycerides were also higher in patients with uncontrolled versus controlled blood pressure (1.9 versus 1.7 mmol/L, *P* < 0.0001). The regional comparison revealed an increase in LDL-cholesterol as well as triglycerides with increasing SBP and DBP for all 5 regions, whereas no association between HDL-cholesterol and blood pressure levels was observed. For details see Tables 6, 7, and 8.

### 3.5. Blood Pressure and CRP

The mean CRP was higher in patients with SBP ≥180 versus <120 mmHg (1.1 versus 0.7 mmol/L), in patients with DBP ≥110 versus <90 mmHg (1.0 versus 0.8 mmol/L), and in patients with uncontrolled versus controlled blood pressure (1.0 versus 0.8 mmol/L) (*P* < 0.0001 for all parameters). An increase in CRP with SBP and DBP was observed in Northern Europe and Northern America only. For details see Table 9.

## 4. Discussion

In the present analysis of a large international study of patients treated for arterial hypertension, both an elevated SBP and DBP, and uncontrolled hypertension were associated with increasing BMI, WC, LDL-cholesterol, triglycerides, HbA1c, and CRP, whereas there was no association between HDL-cholesterol and blood pressure levels. Furthermore, the presence of diabetes was associated with an elevated SBP only. The observed associations between blood pressure levels and metabolic parameters were consistent across all 5 geographic regions, even though some associations were not significant, especially in regions with a low sample size for individual parameters, such as the Middle East, Asia, and—partly—North America. Based on the data presented herein, it appears difficult to draw any firm conclusions on stronger and weaker associations of individual cardiometabolic parameters with blood pressure for some regions as compared to the overall population or the European region. Furthermore, regional samples cannot be necessarily considered as ethnically/culturally homogenous and any regional analysis might be confounded by differences in the genetics or dietary habits of study participants. 

Overall, our data are consistent with findings from other investigations, where the prevalence of additional cardiometabolic risk factors among hypertensive patients was as high as 82% and was associated with poor blood control in the United States [12]. Of interest, data from the large European Global Cardiometabolic Risk Profile in Patients with Hypertension Disease (GOOD) survey in 3280 outpatients treated for or newly diagnosed with hypertension indicate that the prevalence of cardiometabolic risk factors is higher in Central Europe (Hungary) and Atlantic European Mainland (Belgium, Germany, and the Netherlands) compared with the Northwest (Norway, Sweden, and the United Kingdom) and Mediterranean (Italy, Portugal, Slovenia, Spain, and Turkey) regions [13]. Similarly to the GOOD Survey, only one quarter of patients had controlled blood pressure in our study [14]. 

Our results confirm the significant association between systemic hypertension and other cardiometabolic risk factors, including visceral obesity, diabetes, and hyperlipidemia. Obviously, the vast majority of patients with arterial hypertension are at multiple risk of cardiovascular disease. Therefore, our data emphasize the statement of current joint guidelines of the European Society of Hypertension and European Society of Cardiology concerning an intensified diagnostic and therapeutic measures in patients with an elevated SBP and DBP [8]. 

Reasons for the observed association between increasing blood pressure and the presence of cardiometabolic risk factors remain to be determined. It is a subject of an ongoing debate, whether patients with an elevated SBP and DBP simply more frequently have an unfavorable cardiometabolic risk profile with poorly treated cardiovascular parameters or whether there is a causal relationship between a high systemic blood pressure and the deterioration of multiple cardiometabolic markers. The intra-abdominal obesity and recently discovered endogenous gland activity of adipose tissue producing various hormones and cytokines, such as angiotensinogen, insulin, resistin, lipoprotein lipase, leptin, lactate, plasminogen activator inhibitor, adipsin, and interleukin, seem to play a central role in the development of disadvantageous cardiometabolic profile and may represent the causal link between arterial hypertension, atherogenic dyslipidemia, diabetes, thrombosis, and inflammation [15]. This hypothesis is further supported by the mandatory presence of abdominal obesity in the definition of potentially detrimental metabolic syndrome [16, 17]. Other possible reasons include organ damage as a consequence of hypertension which may lead to potentiation of other cardiometabolic risk factors. In addition, visceral obesity, hypertriglyceridemia, and low HDL-cholesterol levels were associated with resistance to antihypertensive therapy in the GOOD survey [18]. 

Proinflammatory mechanisms are thought to be a hallmark of the cardiovascular disease process, notably in disease states such as hypertension. These findings are often exacerbated by the increasing prevalence of obesity worldwide. Obesity is often accompanied by high plasma levels of nonesterified fatty acids that cause insulin resistance in skeletal muscle and overload the liver with lipids, producing fatty liver and atherogenic dyslipidemia [19]. Fat accumulation in the liver may also stimulate hepatic cytokine production and lead to higher levels of proinflammatory markers. Taken together, the abnormal proinflammatory state leads to a worsening of metabolic control, abnormal vascular function, and eventually cardiovascular and renal diseases [20].

Lifestyle changes, including an increased prevalence of obesity and the metabolic syndrome contribute to the incidence of hypertension [21, 22]. At the environmental level, barriers to healthy lifestyles include lack of access to exercise facilities at work or in the community, lack of bicycle and walking paths, and high traffic and crime in urban settings which prevent access to safe walking areas. Seasonal variation, market availability, and affordability of fresh fruits and vegetables in small urban stores are issues, thus multilevel approaches incorporating both individual and policy level changes are advocated. These variations are magnified within certain ethnic and geographical situations. Nevertheless, despite the uncertainty about the causal relationship between an elevated SBP and DBP and the presence of cardiometabolic risk factors, the association appeared to be significant and consistent across various continents and ethnicities in our study. The benefits of a multidimensional approach influencing antioxidative, antiinflammatory, or antithrombotic pathway on cardiovascular outcomes were repeatedly demonstrated in the context of hypertension management [23]. Consequently, a systematic assessment of the global cardiovascular risk and a risk-based approach to antihypertensive therapy shall be mandated in all patients with arterial hypertension. 

### 4.1. Strengths and Limitations of Our Study

The strengths of our study include the prospective enrollment of a large sample of treated hypertensive patients and the collection of detailed information on systemic blood pressure and cardiometabolic parameters. 

One study limitation is the fact that the numbers of enrolled patients differ substantially between the 5 regions. Therefore, the regional comparisons and *P*-values should be interpreted with caution. Neither a uniform methodology nor a central laboratory was used for measurements of blood pressure and cardiometabolic parameters. Thus, differences in region-specific techniques and measurements may have influenced the comparability of results. Another study limitation is the fact that the present analysis of lipid measurements was not adjusted for statin use. However, the analysis was adjusted for age and, therefore, for age-dependent rise of LDL-cholesterol and triglycerides, and indirectly also for statin use because the elderly more often receive statin treatment. Finally, our study was not designed to explore reasons for the observed association between an elevated blood pressure and cardiometabolic risk factors.

## 5. Conclusions

An elevated SBP and DBP, but also uncontrolled hypertension, are associated with an increase in cardiometabolic risk, independently of the geographic region. These findings not only highlight the importance of a thorough risk-stratification of patients with arterial hypertension, but also the necessity of treating concomitant cardiometabolic risk factors in order to decrease the overall cardiovascular risk of patients with arterial hypertension.

## Figures and Tables

**Table 1:** Patient characteristics.

	Total (*N* = 17, 092)	Northern Europe (*N* = 5, 655)	Southern Europe (*N* = 6, 655)	North America (*N* = 1, 455)	Middle East (*N* = 570)	Asia (*N* = 2, 757)
Age	63.1	64.9	62.5	65.7	57.1	60.5
Gender (male, %)	52.8	53.0	52.9	56.3	61.0	48.5
BMI (kg/m²)	28.9	29.7	29.2	30.2	29.8	25.9
Waist circumference (cm)	99.7	102.5	100.9	102.6	102.5	89.5
Systolic blood pressure (mmHg)	148.9	151.5	148.6	144.3	156.6	145.1
Diastolic blood pressure (mmHg)	87.0	87.7	87.7	81.4	92.0	85.6
Uncontrolled blood pressure (%)*	76.3	82.1	75.6	64.9	87.9	69.5
Diabetes mellitus (%)	29.1	33.9	27.4	30.9	33.8	21.7
HbA1c (%)	6.8	6.7	6.7	6.7	7.9	7.1
LDL cholesterol (mmol/L)	3.2	3.2	3.3	2.6	3.4	3.1
HDL cholesterol (mmol/L)	1.3	1.5	1.3	1.3	1.1	1.3
Triglycerides (mmol/L)	1.8	1.9	1.7	1.7	2.0	1.8
Hyperlipidemia (%)	49.0	53.0	43.3	64.4	56.1	46.1
Smoking (current/past; %)	38.8	36.4	41.8	55.7	44.9	28.5
Family history of MI (%)	28.6	22.0	29.6	40.0	25.5	36.3
Microalbuminuria (%)	58.6	54.3	59.6	53.8	71.6	64.7
CRP (mg/dL)	0.92	1.02	0.91	0.54	0.91	0.49
Coronary artery disease (%)	25.1	21.5	23.7	40.5	30.4	26.4
Congestive heart failure (%)	6.4	6.3	6.7	5.5	8.3	6.0
Atrial fibrillation (%)	9.3	9.5	11.1	11.7	4.7	4.0
Myocardial infarction (%)	31.6	24.4	34.1	41.7	27.9	37.1
Ischemic stroke (%)	5.1	24.7	5.5	5.6	4.4	14.6
Peripheral artery disease (%)	4.6	6.1	5.0	5.7	4.7	0.5
Betablockers (%)	48.7	59.7	40.2	44.8	52.5	48.1
Calcium Antagonists (%)	36.0	30.3	31.9	43.4	36.7	53.6
ACE-Inhibitors (%)	42.3	45.8	42.8	49.5	31.9	32.1
AT1-Rezeptorantagonists (%)	35.8	30.1	41.3	31.1	47.9	34.4
Diuretics (%)	9.9	10.9	10.5	8.0	10.7	7.4

*Uncontrolled blood pressure was defined as SBP/DBP ≥140/90 in non-diabetic and ≥130/80 in diabetic patients.

**Table 2:** Blood pressure and BMI (kg/m^2^; mean ± SE; adjusted for age, gender).

	Total (*N* = 16, 945)	Northern Europe (*N* = 5, 621)	Southern Europe (*N* = 6583)	North America (*N* = 1, 423)	Midde East (*N* = 567)	Asia (*N* = 2, 751)	*P* value
SBP (mmHg)							
<120	28.2 (0.249)	29.1 (0.487)	28.4 (0.453)	30.1 (0.601)	28.3 (1.467)	26.2 (0.474)	<0.0001
120–139	28.5 (0.086)	29.1 (0.168)	28.6 (0.132)	30.4 (0.253)	31.0 (0.647)	26.4 (0.187)	<0.0001
140–159	28.8 (0.066)	29.6 (0.108)	29.0 (0.102)	30.2 (0.222)	29.7 (0.370)	25.7 (0.158)	<0.0001
160–179	29.5 (0.105)	30.2 (0.169)	29.9 (0.165)	30.2 (0.403)	29.6 (0.474)	25.7 (0.287)	<0.0001
≥180	29.5 (0.163)	30.2 (0.249)	30.2 (0.264)	29.1 (0.763)	30.0 (0.629)	25.5 (0.445)	<0.0001
*P* value	<0.0001	<0.0001	<0.0001	<0.0001	0.0126	<0.0001	
DBP (mmHg)							
<90	28.5 (0.063)	29.3 (0.110)	28.6 (0.102)	29.9 (0.176)	29.7 (0.440)	26.0 (0.144)	<0.0001
90–99	29.2 (0.082)	30.1 (0.134)	29.4 (0.124)	30.5 (0.342)	29.5 (0.395)	25.7 (0.207)	<0.0001
100–109	29.5 (0.113)	30.0 (0.187)	30.2 (0.169)	31.5 (0.548)	30.4 (0.471)	25.9 (0.276)	<0.0001
≥110	30.3 (0.227)	30.8 (0.362)	31.0 (0.350)	31.9 (1.286)	30.6 (0.793)	26.3 (0.642)	<0.0001
*P* value	<0.0001	<0.0001	<0.0001	<0.0001	0.0157	<0.0001	
RR (mmHg)							
uncontrolled*	29.4 (0.066)	29.9 (0.085)	29.5 (0.082)	30.1 (0.190)	29.8 (0.259)	25.7 (0.132)	<0.0001
controlled**	28.6 (0.061)	28.9 (0.170)	28.4 (0.134)	30.3 (0.239)	30.6 (0.650)	26.2 (0.184)	<0.0001
*P* value	<0.0001	<0.0001	<0.0001	<0.0001	0.0080	<0.0001	

*SBP/DBP ≥140/90 in non-diabetic and ≥130/80 in diabetic patients, **<140/90 in non-diabetic and <130/80 in diabetic patients.

**Table 3:** Blood pressure and WC (cm ± SD, adjusted for age, gender).

	Total (*N* = 16, 808)	Northern Europe (*N* = 5, 568)	Southern Europe (*N* = 6, 505)	North America (*N* = 1, 435)	Middle East (*N* = 553)	Asia (*N* = 2, 747)	*P* value
SBP (mmHg)							
<120	97.5 (0.557)	100.2 (1.028)	98.5 (0.948)	100.9 (1.254)	96.9 (3.078)	89.9 (0.994)	<0.0001
120–139	98.5 (0.218)	100.3 (0.400)	99.2 (0.314)	102.1 (0.600)	100.8 (1.536)	90.6 (0.445)	<0.0001
140–159	99.6 (0.171)	102.2 (0.268)	100.3 (0.255)	102.5 (0.548)	101.0 (0.923)	89.0 (0.392)	<0.0001
160–179	100.9 (0.244)	103.4 (0.373)	102.0 (0.364)	102.4 (0.880)	102.4 (1.070)	89.0 (0.634)	<0.0001
≥180	101.2 (0.414)	103.3 (0.603)	103.4 (0.642)	101.3 (1.816)	104.6 (1.528)	89.2 (1.067)	<0.0001
*P* value	<0.0001	<0.0001	<0.0001	<0.0001	0.1275	<0.0001	
DBP (mmHg)							
<90	98.8 (0.154)	101.1 (0.256)	99.5 (0.237)	101.7 (0.405)	100.1 (1.024)	89.9 (0.334)	<0.0001
90–99	100.1 (0.198)	103.1 (0.309)	100.5 (0.287)	102.5 (0.782)	100.4 (0.924)	89.0 (0.476)	<0.0001
100–109	101.3 (0.305)	102.8 (0.485)	103.4 (0.441)	105.1 (1.417)	105.6 (1.230)	89.5 (0.716)	<0.0001
≥110	103.2 (0.607)	105.1 (0.916)	104.1 (0.891)	107.4 (3.250)	105.0 (2.041)	89.7 (1.611)	<0.0001
*P* value	<0.0001	<0.0001	<0.0001	<0.0001	0.0012	<0.0001	
RR (mmHg)							
uncontrolled*	100.7 (0.167)	102.7 (0.201)	101.2 (0.195)	102.2 (0.445)	102.1 (0.619)	89.2 (0.311)	<0.0001
controlled**	98.8 (0.149)	100.1 (0.400)	99.0 (0.315)	102.1 (0.559)	100.0 (1.523)	90.2 (0.430)	<0.0001
*P* value	<0.0001	<0.0001	<0.0001	<0.0001	0.0882	<0.0001	

*SBP/DBP ≥140/90 in non-diabetic and ≥130/80 in diabetic patients, **<140/90 in non-diabetic and <130/80 in diabetic patients.

**Table 4:** Blood pressure and diabetes (% (95% CI), adjusted for age, and gender).

	Total (*N* = 16, 325)	Northern Europe (*N* = 5, 415)	Southern Europe (*N* = 6, 238)	North America (*N* = 1, 406)	Middle East (*N* = 519)	Asia (*N* = 2, 747)	*P* value
SBP (mmHg)							
<120	28.4 (24.78; 31.94)	28.3 (21.79; 35.96)	33.2 (26.56; 40.53)	36.5 (27.70; 46.41)	32.3 (14.11; 58.00)	21.4 (15.72; 28.48)	<0.0001
120–139	27.3 (25.86; 28.65)	33.7 (30.86; 36.77)	25.7 (23.65; 27.94)	33.6 (29.39; 38.16)	33.9 (23.15; 46.63)	22.7 (19.92; 25.83)	<0.0001
140–159	28.8 (27.69; 29.83)	33.6 (31.77; 35.57)	29.8 (28.12; 31.63)	28.8 (25.21; 32.59)	40.0 (33.26; 47.09)	22.5 (20.07; 25.07)	<0.0001
160–179	30.4 (28.98; 31.89)	37.4 (34.96; 33.96)	31.4 (29.16; 33.84)	32.7 (27.24; 38.59)	32.1 (25.62; 39.44)	21.0 (17.62; 24.84)	<0.0001
≥180	32.6 (30.12; 35.00)	34.8 (31.08; 38.77)	36.5 (32.50; 40.66)	16.4 (9.30; 27.44)	49.5 (38.68; 60.35)	26.8 (20.84; 33.64)	<0.0001
*P* value	<0.0001	0.1571	0.0009	0.0491	0.1353	0.6222	
DBP (mmHg)							
<90	30.9 (29.95; 31.95)	37.6 (35.77; 39.53)	29.6 (27.97; 31.25)	34.3 (31.51; 37.31)	38.8 (31.44; 46.82)	25.8 (23.58; 28.08)	<0.0001
90–99	27.9 (26.76; 29.14)	34.2 (32.11; 36.36)	29.2 (27.32; 31.08)	25.3 (20.77; 30.55)	34.4 (28.23; 41.10)	21.2 (18.53; 24.24)	<0.0001
100–109	25.5 (23.70; 27.26)	26.9 (23.92; 30.13)	31.0 (28.16; 34.07)	22.2 (14.84; 31.73)	38.7 (30.21; 47.89)	13.6 (10.43; 17.64)	<0.0001
≥110	28.1 (24.68; 31.55)	31.3 (26.06; 37.12)	33.3 (28.08; 38.99)	18.4 (7.02; 40.17)	42.7 (29.81; 56.68)	16.7 (9.95; 26.69)	<0.0001
*P* value	<0.0001	<0.0001	0.3635	0.0002	0.8877	<0.0001	
RR (mmHg)							
uncontrolled*	27.7 (26.73; 28.71)	34.7 (33.30; 36.06)	30.8 (29.48; 32.07)	28.6 (25.81; 31.56)	38.0 (33.68; 42.54)	22.4 (20.55; 24.34)	<0.0001
controlled**	30.4 (29.42; 31.34)	34.3 (31.37; 37.33)	27.1 (27.98; 29.40)	35.4 (31.36; 39.70)	37.1 (25.98; 49.82)	22.9 (20.18; 25.96)	<0.0001
*P* value	0.1783	0.8316	0.0497	0.0106	0.6824	0.6820	

*SBP/DBP ≥140/90 in non-diabetic and ≥130/80 in diabetic patients, **<140/90 in non-diabetic and <130/80 in diabetic patients.

**Table 5:** Blood pressure and HbA1c (% ± SD, adjusted for age, gender, and diabetes treatment).

	Total (*N* = 3, 582)	Northern Europe (*N* = 1, 640)	Southern Europe (*N* = 1, 058)	North America (*N* = 358)	Middle East (*N* = 149)	Asia (*N* = 377)	*P* value
SBP (mmHg)							
<120	6.7 (0.115)	6.2 (0.202)	6.4 (0.199)	6.6 (0.214)	6.4 (0.725)	6.8 (0.257)	0.4127
120–139	6.6 (0.047)	6.4 (0.072)	6.3 (0.081)	6.4 (0.113)	7.3 (0.348)	6.7 (0.114)	0.0073
140–159	6.8 (0.037)	6.5 (0.049)	6.5 (0.062)	6.6 (0.111)	7.3 (0.168)	6.8 (0.102)	<0.0001
160–179	6.9 (0.052)	6.6 (0.063)	6.7 (0.084)	6.6 (0.167)	7.6 (0.187)	6.9 (0.179)	<0.0001
≥180	7.0 (0.084)	6.7 (0.102)	6.9 (0.135)	6.4 (0.402)	8.0 (0.243)	6.9 (0.292)	<0.0001
*P* value	<0.0001	0.0003	0.0012	0.4751	0.6808	0.7181	
DBP (mmHg)							
<90	6.8 (0.031)	6.5 (0.043)	6.4 (0.055)	6.5 (0.074)	7.2 (0.172)	6.8 (0.080)	<0.0001
90–99	6.8 (0.045)	6.5 (0.056)	6.5 (0.069)	6.6 (0.175)	7.6 (0.180)	6.7 (0.128)	<0.0001
100–109	6.9 (0.076)	6.6 (0.096)	6.8 (0.111)	6.4 (0.312)	8.0 (0.226)	6.4 (0.243)	<0.0001
≥110	6.9 (0.172)	6.7 (0.211)	6.6 (0.288)	7.7 (0.934)	7.2 (0.489)	7.2 (0.921)	0.6603
*P* value	0.0027	0.1605	0.0136	0.3190	0.3332	0.4885	
RR (mmHg)							
uncontrolled*	6.8 (0.039)	6.5 (0.035)	6.6 (0.046)	6.6 (0.088)	7.6 (0.111)	6.8 (0.082)	<0.0001
controlled**	6.7 (0.030)	6.3 (0.073)	6.3 (0.079)	6.5 (0.104)	7.1 (0.316)	6.7 (0.110)	0.0075
*P* value	<0.0001	0.0004	0.0091	0.2444	0.3367	0.3114	

*SBP/DBP ≥140/90 in non-diabetic and ≥130/80 in diabetic patients, **<140/90 in non-diabetic and <130/80 in diabetic patients.

**Table 6:** Blood pressure and LDL-Cholesterol (mmol/L ± SD, adjusted for age, gender, and diabetes).

	Total (*N* = 11,529)	Northern Europe (*N* = 3, 723)	Southern Europe (*N* = 4, 679)	North America (*N* = 904)	Middle East (*N* = 485)	Asia (*N* = 1, 738)	*P* value
SBP (mmHg)							
<120	2.9 (0.044)	3.0 (0.088)	3.0 (0.076)	2.5 (0.109)	2.8 (0.231)	2.8 (0.093)	0.0003
120–139	3.0 (0.048)	3.1 (0.035)	3.0 (0.028)	2.4 (0.054)	2.9 (0.121)	2.9 (0.042)	<0.0001
140–159	3.2 (0.015)	3.2 (0.025)	3.2 (0.024)	2.6 (0.053)	3.4 (0.075)	3.0 (0.038)	<0.0001
160–179	3.3 (0.020)	3.3 (0.033)	3.3 (0.032)	2.7 (0.081)	3.5 (0.080)	3.3 (0.057)	<0.0001
≥180	3.4 (0.034)	3.4 (0.055)	3.5 (0.058)	3.0 (0.153)	3.9 (0.131)	3.5 (0.096)	<0.0001
*P* value	<0.0001	<0.0001	<0.0001	<0.0001	0.0005	<0.0001	
DBP (mmHg)							
<90	3.0 (0.013)	3.1 (0.022)	3.1 (0.021)	2.4 (0.037)	3.0 (0.081)	2.9 (0.031)	<0.0001
90–99	3.3 (0.016)	3.3 (0.028)	3.3 (0.027)	2.8 (0.074)	3.4 (0.073)	3.1 (0.045)	<0.0001
100–109	3.4 (0.026)	3.4 (0.047)	3.4 (0.041)	2.9 (0.141)	3.9 (0.098)	3.4 (0.069)	<0.0001
≥110	3.5 (0.049)	3.5 (0.086)	3.6 (0.080)	3.9 (0.311)	3.5 (0.169)	3.6 (0.151)	0.5736
*P* value	<0.0001	<0.0001	<0.0001	<0.0001	<0.0001	<0.0001	
RR (mmHg)							
uncontrolled*	3.4 (0.014)	3.3 (0.019)	3.3 (0.018)	2.7 (0.042)	3.5 (0.050)	3.1 (0.029)	<0.0001
controlled**	3.0 (0.012)	3.0 (0.034)	3.0 (0.027)	2.4 (0.050)	2.9 (0.115)	2.9 (0.040)	<0.0001
*P* value	<0.0001	<0.0001	<0.0001	<0.0001	<0.0001	<0.0001	

*SBP/DBP ≥140/90 in non-diabetic and ≥130/80 in diabetic patients, **<140/90 in non-diabetic and <130/80 in diabetic patients.

**Table 7:** Blood pressure and HDL-Cholesterol (mmol/L ± SD, adjusted for age, gender, and diabetes).

	Total (*N* = 11, 849)	Northern Europe (*N* = 3, 787)	Southern Europe (*N* = 4, 924)	North America (*N* = 909)	Middle East (*N* = 477)	Asia (*N* = 1, 752)	*P* value
SBP (mmHg)							
<120	1.3 (0.025)	1.4 (0.051)	1.2 (0.044)	1.4 (0.065)	1.1 (0.135)	1.3 (0.053)	0.0498
120–139	1.3 (0.008)	1.4 (0.015)	1.3 (0.012)	1.3 (0.024)	1.1 (0.054)	1.3 (0.018)	<0.0001
140–159	1.3 (0.007)	1.5 (0.011)	1.3 (0.010)	1.3 (0.024)	1.2 (0.034)	1.3 (0.017)	<0.0001
160–179	1.3 (0.009)	1.4 (0.014)	1.3 (0.014)	1.3 (0.035)	1.1 (0.035)	1.3 (0.025)	<0.0001
≥180	1.3 (0.016)	1.5 (0.025)	1.2 (0.026)	1.3 (0.070)	1.0 (0.059)	1.3 (0.045)	<0.0001
*P* value	0.3309	0.0558	0.1317	0.3366	0.6608	0.7711	
DBP (mmHg)							
<90	1.3 (0.005)	1.4 (0.010)	1.3 (0.009)	1.3 (0.017)	1.2 (0.038)	1.3 (0.014)	<0.0001
90–99	1.3 (0.007)	1.4 (0.012)	1.3 (0.011)	1.3 (0.033)	1.2 (0.033)	1.3 (0.020)	<0.0001
100–109	1.3 (0.011)	1.4 (0.016)	1.2 (0.014)	1.3 (0.047)	1.1 (0.034)	1.2 (0.024)	<0.0001
≥110	1.3 (0.033)	1.6 (0.057)	1.2 (0.052)	1.4 (0.208)	1.0 (0.114)	1.2 (0.102)	<0.0001
*P* value	0.0222	0.0013	0.0904	0.7721	0.0188	0.6265	
RR (mmHg)							
uncontrolled*	1.3 (0.006)	1.5 (0.008)	1.3 (0.008)	1.3 (0.019)	1.1 (0.022)	1.3 (0.013)	<0.0001
controlled**	1.3 (0.006)	1.4 (0.016)	1.3 (0.013)	1.3 (0.023)	1.1 (0.054)	1.3 (0.019)	<0.0001
*P* value	0.1340	0.0916	0.1632	0.9361	0.8361	0.8030	

*SBP/DBP ≥140/90 in non-diabetic and ≥130/80 in diabetic patients, **<140/90 in non-diabetic and <130/80 in diabetic patients.

**Table 8:** Blood pressure and triglycerides (mmol/L ± SD, adjusted for age, gender, and diabetes).

	Total (*N* = 12, 601)	Northern Europe (*N* = 4, 049)	Southern Europe (*N* = 5, 095)	North America (*N* = 910)	Middle East (*N* = 504)	Asia (*N* = 2043)	*P* value
SBP (mmHg)							
<120	1.5 (0.037)	1.6 (0.078)	1.6 (0.068)	1.5 (0.102)	1.3 (0.206)	1.7 (0.076)	0.4212
120–139	1.7 (0.017)	1.8 (0.033)	1.6 (0.027)	1.8 (0.054)	1.7 (0.120)	1.8 (0.038)	<0.0001
140–159	1.8 (0.014)	1.9 (0.025)	1.7 (0.023)	1.7 (0.054)	2.1 (0.075)	1.9 (0.036)	<0.0001
160–179	1.9 (0.020)	2.0 (0.033)	1.8 (0.033)	1.7 (0.085)	2.2 (0.082)	2.0 (0.055)	<0.0001
≥180	1.9 (0.034)	2.0 (0.055)	1.8 (0.057)	1.9 (0.157)	2.3 (0.134)	2.2 (0.097)	0.0054
*P* value	<0.0001	<0.0001	<0.0001	0.1658	0.0039	0.0008	
DBP (mmHg)							
<90	1.7 (0.012)	1.8 (0.021)	1.6 (0.020)	1.7 (0.037)	1.7 (0.078)	1.8 (0.028)	<0.0001
90–99	1.9 (0.016)	2.0 (0.028)	1.8 (0.026)	1.9 (0.074)	2.2 (0.073)	1.9 (0.042)	<0.0001
100–109	1.9 (0.027)	2.0 (0.050)	1.8 (0.044)	2.0 (0.154)	2.3 (0.108)	2.1 (0.073)	<0.0001
≥110	1.9 (0.053)	1.8 (0.089)	1.9 (0.084)	1.9 (0.342)	2.4 (0.180)	2.3 (0.161)	0.0073
*P* value	<0.0001	<0.0001	<0.0001	0.0469	0.0012	<0.0001	
RR (mmHg)							
uncontrolled*	1.9 (0.014)	2.0 (0.018)	1.7 (0.017)	1.8 (0.043)	2.1 (0.050)	1.9 (0.028)	<0.0001
controlled**	1.7 (0.011)	1.7 (0.033)	1.6 (0.027)	1.7 (0.050)	1.7 (0.115)	1.8 (0.037)	0.0024
*P* value	<0.0001	<0.0001	<0.0001	0.4255	0.0025	0.0081	

*SBP/DBP ≥140/90 in non-diabetic and ≥130/80 in diabetic patients, **<140/90 in non-diabetic and <130/80 in diabetic patients.

**Table 9:** Blood pressure and CRP (mg/dL ± SD, adjusted for age, and gender).

	Total (*N* = 2, 493)	Northern Europe (*N* = 1, 207)	Southern Europe (*N* = 943)	North America (*N* = 109)	Middle East (*N* = 112)	Asia (*N* = 122)	*P* value
SBP (mmHg)							
<120	0.7 (0.090)	0.8 (0.131)	0.8 (0.160)	0.6 (0.284)	0.6 (0.576)	0.1 (0.304)	0.3399
120–139	0.7 (0.037)	0.7 (0.062)	0.8 (0.053)	0.4 (0.154)	0.4 (0.286)	0.4 (0.139)	0.0141
140–159	0.9 (0.030)	1.0 (0.043)	0.9 (0.050)	0.5 (0.146)	1.1 (0.145)	0.5 (0.133)	0.0009
160–179	1.1 (0.041)	1.1 (0.055)	1.1 (0.070)	0.5 (0.250)	0.9 (0.168)	0.6 (0.219)	0.0196
≥180	1.1 (0.066)	1.3 (0.087)	0.8 (0.117)	1.1 (0.301)	0.8 (0.230)	0.3 (0.500)	0.0097
*P* value	<0.0001	<0.0001	0.0520	0.0356	0.4130	0.3783	
DBP (mmHg)							
<90	0.8 (0.027)	0.8 (0.038)	0.8 (0.043)	0.4 (0.112)	0.9 (0.164)	0.4 (0.114)	<0.0001
90–99	1.0 (0.035)	1.1 (0.049)	0.9 (0.058)	0.6 (0.187)	1.1 (0.150)	0.6 (0.155)	0.0022
100–109	1.1 (0.050)	1.3 (0.072)	1.0 (0.078)	1.0 (0.293)	0.8 (0.193)	0.4 (0.253)	0.0013
≥110	1.0 (0.091)	1.2 (0.130)	0.8 (0.158)	0.6 (0.424)	0.9 (0.338)	0.9 (0.456)	0.3130
*P* value	<0.0001	<0.0001	0.4665	0.0056	0.5488	0.1511	
RR (mmHg)							
uncontrolled*	1.0 (0.028)	1.1 (0.031)	0.9 (0.037)	0.6 (0.114)	0.9 (0.096)	0.5 (0.106)	<0.0001
controlled**	0.8 (0.026)	0.7 (0.061)	0.8 (0.054)	0.4 (0.143)	0.6 (0.348)	0.4 (0.140)	0.0057
*P* value	<0.0001	<0.0001	0.1596	0.2786	0.4071	0.1582	

*SBP/DBP ≥140/90 in non-diabetic and ≥130/80 in diabetic patients, **<140/90 in non-diabetic and <130/80 in diabetic patients.
